# The Wellcome Trust/Burroughs Wellcome Fund joint program in infectious diseases of the tropical developing world.

**DOI:** 10.3201/eid0707.017737

**Published:** 2001

**Authors:** V McGovern

**Affiliations:** Burroughs Wellcome Fund, Research Triangle Park, North Carolina 27709-3901, USA. vmgovern@bwfund.org


### The Wellcome Trust/Burroughs Wellcome Fund Joint Program in Infectious Diseases of the Tropical Developing World

**Victoria McGovern**
Burroughs Wellcome Fund, Research Triangle Park, North Carolina, USA

The Burroughs Wellcome Fund is a U.S.-based, private, independent foundation whose mission is to advance the medical sciences by supporting research and other scientific and educational activities. As a medium-sized foundation with assets of approximately $730 million, the fund invests $30 to $35 million in research each year, primarily in the United States and Canada. The foundation has been supporting basic research in infectious diseases, especially parasitic diseases, since 1981.

Since the Burroughs Wellcome Fund is relatively small, we must leverage research investments, making sure that our modest funds can have an impact beyond their face value. One way of increasing the value of our investment is to establish partnerships with other organizations. This approach allows funders to extend their support to new areas without diverting large parts of their limited resources from currently funded areas.

In 1997, the Board of Governors of the Wellcome Trust, the world's largest medical research charity, set aside $25 million to fund a 2-year collaborative venture with the Burroughs Wellcome Fund. Through this collaboration with the Wellcome Trust,[1] the Burroughs Wellcome Fund has been able to enter the international health arena. In 2000, the fund's board of directors added $1 million to the program, a sum proportionate to the trust's $25 million investment, based on each organization's respective annual research spending.

The shared program, a jointly administered effort focusing on infectious diseases in the tropical developing world, supports research collaborations between workers in the United Kingdom, North America, and the tropics. Research funded by the program must focus on diseases especially important in the developing world. It must involve a true three-way collaboration in which all the partners play interdependent and supportive roles. Importantly, it requires that the collaboration's "center of gravity" be in the tropics: the tropical world must truly contain the lynchpin of the project and not simply be the collecting ground for interesting problems to be taken away and studied elsewhere. The aim is to build true partnerships that benefit all collaborators and enhance the research strength of the developing world partner.

The problem of "brain drain" from the developing world to the developed world is significant, and it will not be solved with small investments. By strengthening collaborations and focusing on developing world research problems in situ, investors can enhance the strength of developing world scientists and stimulate further investment as their work unfolds.

The Burroughs Wellcome Fund will use the results of this program to study the fund's potential roles in this arena. The need for world health funding is vast and daunting, especially for smaller funders, but support of basic research and stimulation of research collaboration can be done with far fewer dollars than other approaches to world health.

The joint program's second and final round concluded in late 2000 with selection of six new awardee programs, in addition to the seven programs supported in 1999.

Address for correspondence: Victoria McGovern, Burroughs Wellcome Fund, 21 T.W. Alexander Drive, P.O. Box 13901, Research Triangle Park, NC 27709-3901, USA; fax: 919-991-5172; e-mail: vmcgovern@bwfund.org

[1] These two organizations have related names and some shared history but are completely independent of one another. The trust focuses its funding in the United Kingdom, but also supports a large international research program. In general, the trust does not fund projects in the United States or Canada.

